# Visual to Parametric Interaction (V2PI)

**DOI:** 10.1371/journal.pone.0050474

**Published:** 2013-03-20

**Authors:** Scotland C. Leman, Leanna House, Dipayan Maiti, Alex Endert, Chris North

**Affiliations:** 1 Department of Statistics, Virginia Tech, Blacksburg, Virginia, United States of America; 2 Department of Computer Science, Virginia Tech, Blacksburg, Virginia, United States of America; University of East Piedmont, Italy

## Abstract

Typical data visualizations result from linear pipelines that start by characterizing data using a model or algorithm to reduce the dimension and summarize structure, and end by displaying the data in a reduced dimensional form. Sensemaking may take place at the end of the pipeline when users have an opportunity to observe, digest, and internalize any information displayed. However, some visualizations mask meaningful data structures when model or algorithm constraints (e.g., parameter specifications) contradict information in the data. Yet, due to the linearity of the pipeline, users do not have a natural means to adjust the displays. In this paper, we present a framework for creating dynamic data displays that rely on both mechanistic data summaries and expert judgement. The key is that we develop both the theory and methods of a new human-data interaction to which we refer as “ Visual to Parametric Interaction” (V2PI). With V2PI, the pipeline becomes bi-directional in that users are embedded in the pipeline; users learn from visualizations and the visualizations adjust to expert judgement. We demonstrate the utility of V2PI and a bi-directional pipeline with two examples.

## Introduction

Organizing and understanding large datasets are complex tasks for many scientists, engineers, and intelligence analysts. To aid them in such sensemaking endeavors, tools have been developed to visualize high-dimensional data. These tools rely on mathematical models or algorithms that collapse high-dimensional data matrices to much smaller visual spaces (i.e., spaces of only two or three dimensions). For example, common visualizations of high-dimensional text data extend upon a geography metaphor and use algorithms to display such data in two-dimensional maps [1]. One problem is that visualizations can mislead users, just as any data summary might, by over-simplifying features or structures in high-dimensional datasets. Therefore, low-dimensional versions of high-dimensional data have the potential to be misleading. When this happens, users currently have limited options to correct the problem.

**Figure 1:**
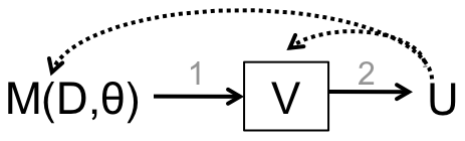
Standard visualization pipeline. Data 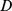 feeds into a mathematical model 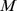 that relies also on parameters 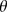, and produces a visualization 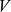. The users 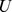 make sense of the visualization to the best other their abilities. To correct any visual inaccuracies, users must either change 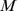, 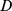, or 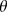.

Namely, displays of data in two or three dimensions result typically from a linear visualization pipeline shown in Figure 1, where data 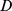 are summarized by a mathematical model or algorithm 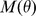 first and subsequently mapped to a visual display 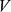. A display is controlled solely by the algorithm that generated it and adheres to predefined mathematical objectives, constraints, or parameters denoted by 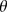. When these constraints contradict expert judgment, they warp or miss useful data features and visualizations can lose interpretability. For examples, consider visualization methods Principal Component Analysis (PCA) [2] and Multidimensional Scaling (MDS) [3]. PCA is a common analytical approach that projects datasets to two dimensions (in the case of visualization) in the directions with the highest variance. PCA loses its utility when meaningful features in the data do not correspond with variance. Similarly, MDS is an analytical approach that solves for low-dimensional (e.g., two-dimensional) coordinates of data points by minimizing the difference between pairwise distances of observations in high- and low-dimensional spaces. When the the chosen distance function lacks relevance to the application, MDS can produce visualizations that are hard to interpret.

As defined by the current pipeline (Figure 1), users do not have an intuitive or natural means to correct visual inaccuracies-beyond the option of starting the pipeline over. For example, users can transform the data or adjust the display-generating model (e.g., tweak model parameters 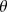) to re-implement the pipeline and create new visualizations. This means that users, who may not have the appropriate mathematical training, must have a deep enough understanding of the display-generating models to change them or the data in a way that will result in useful visualizations. When users cannot parameterize their expert judgements, the pipeline is broken and sensemaking stalls.

In the field of Visual Analytics (VA), the disconnect between static displays of data and usability has been studied extensively and has motivated research in human-computer interaction [4]–[7]. It has been shown that when users interact with visualizations, users learn more from the data than when they do not, even when the model is arguably poor. In the Methods Section, we define common forms of interaction that are readily available in many VA tools: surface-level and parametric interaction. VA tools that enable surface-level interactions allow users to edit displays independent of the underlying models or algorithms; e.g., high-lighting or filtering observations. Whereas, when users interact parametrically, they manually change influential parameters in the display-generating models or algorithms. For example, iPCA [8] and XGvis [9] are VA tools that allow users to adjust dials or sliders that either augment or out-right change influential parameters in PCA or MDS, respectively.

Despite the extensive development of human-computer interactions and VA tools, sensemaking of complex data is still limited. The pitfall of current forms of interactions is that users are still constrained by the linear pipeline and placed at either the beginning or end. For example, surface-level interactions take place at the end of the pipeline by users who are trying to salvage information from a potentiality misleading view of the data. Whereas, parametric interactions take place at the beginning of the pipeline by users who are forced to make model adjustments. Although parameter-controlling dials and sliders are easy to manipulate, users must still understand the mathematical models or algorithms to explore data efficiently. Without understanding the mathematics, users have two options a) hope (not know) that their parametric adjustments will convey their expert judgments appropriately or b) evaluate data visualizations given *every* possible combination of the parametric settings. For one parameter (e.g., one dial), option b) is do-able, even when the parameter is continuous. However, given two or more continuous parameters, we think that option b) will quickly overwhelm users by the infinite number of parameter combinations.

In this paper, we discuss a new form of human-computer interaction to which we refer as “Visual to Parametric Interaction” (V2PI). We recognized that when users make certain surface-level changes to displays, the users are communicating that the display-generating algorithm is not working properly. V2PI s interpret quantitatively what is communicated by the users to make parametric model changes (and, subsequently, new visualizations). In Endert et al. [10], we provide specific examples of V2PI. Here, we not only apply V2PI, but also define V2PI explicitly, highlight the framework to develop a V2PI, discuss advantages and disadvantages of V2PI, and explain fundamental changes in the process to visualize and explore data when V2PI is possible. V2PI transforms data visualizations from being static to dynamic in that V2PI enables information to flow fluidly between display-generating algorithms and users. Users learn from visualizations and the visualizations adjust to expert judgement. Thus, with V2PI, the visualization pipeline becomes bi-directional in that users are not simply at the starting nor receiving end of the pipeline, but are embedded in the visualization scheme formally.

To be clear, VA tools that enable V2PI and rely on the bi-directional visualization pipeline foster data exploration. V2PI does not guarantee the discovery of all or any particular feature in the data. As a data exploration tool, V2PI merges two learning technologies: 1) statistical/data mining methods and 2) interactive visualization techniques. The first technology focuses on mathematical/algorithmic representations of data, whereas the second provides cognitive representations of data. While V2PI maintains the rigor of mathematical/algorithmic technologies, users only operate within visual layouts of data. Hence, again, the methodology we develop from the merger is one for data exploration. In our examples (Results Section) we reiterate the exploratory nature of our methods, but for explanatory reasons we offer a “ground-truth” to exemplify how visualizations adjust and what we can learn using V2PI.

The remainder of the paper has four main sections: Methods, Results, Discussion, and Conclusion. In the Methods Section, we provide background about visual analytic interactions and introduce V2PI. We define V2PI, develop the bi-directional visualization pipeline, and explain required steps to construct V2PI VA tools. In the Results Section, we apply V2PI in two case studies. For each case study, we describe the data at hand, a reasonable method for visualizing it, potential feature-masking constraints of the methods, and implement V2PI to relax those constraints. We reflect on the case studies in the Discussion Section to acknowledge both the benefits and limitations of V2PI. In the Conclusion Section, we summarize our current and future work.

## Methods

### 2.1 Background: Visual Analytic Interactions

The process of using data to update domain specific knowledge is referred to as sensemaking [11], [12] and has been represented in the form of a sensemaking process [13], [14]. In this process, analysts (i.e., experts, users, applied researchers, etc.) begin with a knowledge base that they hope to either expand or adjust given the data. The information discernible in data is often unclear to analysts. Thus, learning from data may take place over time or a series iterations during which analysts explore the data and assimilate what they observe with their knowledge bases. Such explorations/assimilations may take place each time analysts interact with data.

In fact, Pike et al. [7] states, “interaction *is* the insight,” and according to Thomas and Cook [12], Visual Analytics (VA) “is the science of analytical reasoning facilitated by interactive visual interfaces.” In VA, various types of interactions have been studied, and Pike et al. [7] categorize them into two main groups: *lower-* and *higher-* level interactions. The primary difference between these groups pertains to the goal of the users when they interact with the data. With lower-level interactions, users aim to summarize “low-level structure” in the data including maxima, minima, simple patterns, and linear trends. Examples of such interactions include filtering, sorting, and other specific formal queries. Any interactions that are not considered lower-level are higher-level. The purpose of higher-level interactions is to “understand” the data by uncovering features based on abstract or complex (e.g., nonlinear) data characterizations.

**Figure 2:**
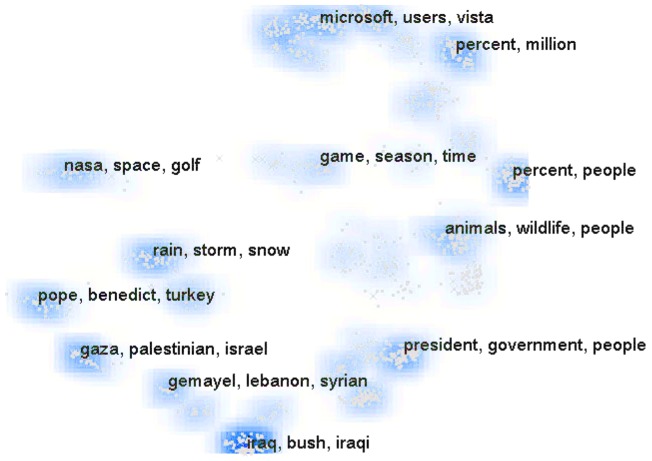
A “galaxy view” of text data created by the IN-SPIRE suite of data visualizations. In-SPIRE uses complex mathematical models in order to discern structure (e.g., clusters) in high-dimensional data.

In this section, we refine the interaction groups further as *surface-level* and *parametric* to motivate the development of V2PI. We explain each the interactions within the context of Figure 2. Figure 2 was created by a VA tool called IN-SPIRE [15] and displays a “Galaxy View” of text data that were collected for an intelligence analysis. In this spatialization, the data points, i.e., documents, are represented by dots and clustered algorithmically by IN-SPIRE. The aim for IN-SPIRE is to assist users in grouping similar documents together and displaying them in an accessible fashion.

#### 2.1.1 Surface-Level Interactions

Surface-level interactions are performed purely within the visual domain and are contained in the lower-level class of interactions. Data rotations, reflections, and translations, highlighting or editing observations, and zooming into a portion of the visual space are each examples of surface-level interactions. These interactions, while capable of enhancing the understanding of complex data structures, do not necessarily relate coherently to mathematical data structures. Within the context of Figure 2, surface-level interactions may include opening, closing, highlighting, and filtering documents or repositioning clusters. For example, users may wish to drag the cluster labeled by, “rain, snow, storm,” to the bottom right of the screen because they feel that the cluster is unimportant. This adjustment is independent of the underlying algorithm and committed purely for organizational purposes.

#### 2.1.2 Parametric Interactions

Parametric interactions are performed directly on the mathematical models that control visualizations. iPCA and XGvis are VA tools that permit parametric interactions; iPCA allows users to interact directly with the principle eigenspace of the data, and XGvis enables users to change either the analytical metric scaling method (measure for distance between observations) or the local optimization scheme used to solve for lower dimensional versions of high-dimensional observations. If IN-SPIRE had the capability for a user to specify, say, the number of clusters in Figure 2, it would be an example of a tool that also permits parametric interactions. Table 1 provides a non-exhaustive list of other parametric interactions.

**Table 1:** A non-exhaustive list of parametric interactions.

Visualization	Parametric Interactions
Data in clusters	A user defines a cluster by specifying the required shape, minimum distance from other clusters, or minimum number of elements.
Data network	A user adjusts the number of nodes and/or edges.
Classification tree diagram	A user adjusts the probabilities that branches split.

Regardless of whether users apply parametric or surface-level interaction, they are often trying to match the visualization to their personal mental maps of the data. A user is more likely to make sense of the data when the data appear in an expected form. However, editorial changes to visualizations dismiss their mathematically driven interpretations, and, parametric changes may not produce ideal visualizations for users. Mental maps of data may not comply to rigid, parametric characterizations of the data. This means that regardless of how many times parameters are adjusted, it is possible that suitable images of data may never be obtained by users. What users need is an interaction that balances surface-level and parametric adjustments to displays of data. For this reason, we develop Visual to Parametric Interactions (V2PI)

### 2.2 Visual to Parametric Interactions (V2PI)

Surface-level interactions are intuitive to implement, but may lack analytical interpretation because they are independent of the mathematical underpinnings of visualizations. Parametric interactions maintain the integrity of mathematical data characterizations, but can be difficult for analysts to understand. To combine the ease of surface-level interactions and the mathematical rigor of parametric interactions, we introduce Visual to Parametric Interaction (V2PI). In this section, we define Visual to Parametric Interaction (V2PI), show how it fits in a bi-directional visualization pipeline, and refine technical points about V2PI. Subsequently, in the Results and Discussion Sections respectively, we apply and summarize V2PI in case studies.

#### 2.2.1 Definition

V2PI is the act of making surface-level interactions that are interpreted by software quantitatively to make parametric model changes (and, subsequently, new visualizations). For example, one interpretation of the clustering structure in Figure 2 is that observations within clusters are more correlated than observations between clusters. Suppose a user chose to commit a surface-level interaction by merging two neighboring clusters together. This interactions suggests that the algorithm (as parameterized) underlying the IN-SPIRE visualization under estimates the correlation between a subset (those selected) or all observations. If IN-SPIRE had V2PI capabilities, IN-SPIRE would quantify and parametrize the merger to adjust all or a subset of pairwise correlation measurements. In turn, IN-SPIRE would use the adjusted correlation measurements to create a new display with different clusters and ready for further V2PI.

The novelty of V2PI is that developers of VA tools with V2PI functionality must learn users' intent from surface-level interactions and develop a strategy to automate mathematical adjustments to display-generating models accordingly. Thus, developers must know, in advance, how to interpret, process, and parametrize various surface-level interactions. Table 2 provides a non-comprehensive list of surface-level interactions with possible parametric interpretations. Alas, not every surface-level interaction will have a meaningful parametric interpretation and, for those that do, the process to parameterize the surface-level interaction is model specific. We discuss the process in the Section 2.2.3, Parameterizing Feedback, and provide examples in the Results Section.

**Table 2:** A non-exhaustive list of V2PI.

Visualization	Surface-Level Interaction	A Parametric Interpretation
Data in clusters	Move two points from different clusters to the same cluster	Up weight the current clustering role of the dimensions in which the observations are similar
Two-dimensional map or spatialization of data	Change the relative locations of points	Down weight the dimensions that dictate the current map
Data network across nodes/data points	Delete a connection between nodes	Decrease the current correlation between the nodes
Classification tree diagram	Delete a classification bra 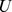 nch	Reduce the current marginal probability of belong to the corresponding class

V2PI requires parametric interpretations of surface-level interactions.

The primary advantage of V2PI is that displays of data that were once static become dynamic. They can respond indefinitely to surface-level interactions (with the parametric interpretations) to account for expert judgment and potentially reveal additional information in the data. Thus, as users learn more, new visualizations can update accordingly; and, as visualizations update, users can learn more. With V2PI, is a bi-directional flow of information in the visual domain of the data between display-generating models and users. In the next section, we develop the concept of a bi-directional visualization pipeline in detail and further explain V2PI.

#### 2.2.2 V2PI and the Bi-directional Pipeline

By construction, visualizations that result after a user's V2PI are dynamic and represent both the high-dimensional data according to the model or algorithm and expert judgement. Users learn from the visualizations and the visualizations adjust to user feedback, as defined by the parametric interpretation of some surface-level interactions. By interpreting the interactions in a parametric form, a) the models or algorithms work as defined originally, but now rely on both the data and user feedback; and b) the models create new visualizations that are subsequently available for additional feedback.

**Figure 3:**
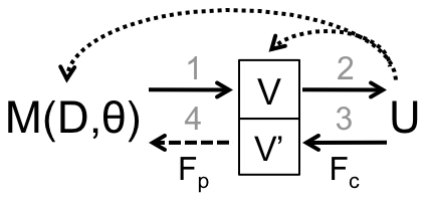
The bi-directional visualization pipeline. Step 1) Create visualization 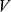 based on a mathematical model or algorithm 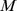 that depends on data 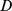 parameters 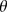; Step 2) display the visualization for users 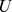 to assess, Step 4) Users adjust the visualization to offer model feedback; and Step 5) Update the model 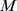 (e.g., via the parameters 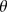).

To see this, consider Figure 3, a bi-directional version of the original visualization pipeline. This version is similar to Figure 1, except users may now receive and distribute information in the visualization iteratively. Specifically, **Steps 1** and **2** of the bi-directional pipeline are similar to the original in that a mathematical model 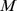 that relies on data 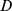 and parameters 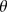 constructs a visualization 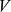 that users 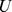 assess for sensemaking. Now, with V2PI, users have the opportunity to commit either standard (surface-level or parametric) interactions or offer feedback about the model via the visualization. If users choose V2PI, they make surface-level adjustments to visualization 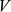 to create 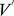 (the original 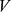 with adjustments). We distinguish standard surface-level interactions from those associated with V2PI by referring to the latter as *cognitive feedback*, 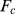. That is, in **Step 3** of the bi-directional pipeline, users communicate their 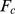 by creating visualization 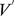. In **Step 4**, the cognitive feedback is parameterized to update 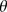 for 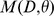 accordingly; we refer to the parameterized version of 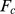 as *parametric feedback* 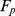. This step is represented by a dashed line because, in practice, users are protected from the parameterization of 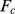. VA developers of visualization tools with V2PI capabilities must have the computational and mathematical machinery in place to parametrize cognitive feedback. Given 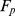 and the updated 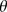, the pipeline steps may repeat.

Steps 1–4 may iterate until any of the following occurs: users are satisfied with the display; the data have been explored thoroughly; or the sensemaking process is complete. For this reason, the bi-directional pipeline is similar in spirit to typical depictions of sensemaking and human-computer interaction, including those developed by Norman, Abowd and Beale, and Keim et al. [5], [16], [17]. Such depictions outline actions that need to be taken by the user and/or the system (e.g., data analysis, computer, visualization, etc.) to enable sensemaking of data. The bi-directional pipeline, however, is more detailed than these depictions. For example, Keim et al. [5] present an iterative “Visual Analytics Process” that considers the potential for users to obtain insight from visualizations and commit interactions “to refine parameters in the analysis process and steer visualization.” Whereas, the bi-directional pipeline describes specifically how users interact with displays of data and how the system interprets these interactions to update the analytical process, when the parametrization machinery (Step 4) is in place. Also, communication between the system and the users in the bi-directional pipeline takes place explicitly in the data visual domain. In fact, visualizations 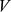 and 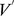 are connected in Figure 3 to emphasize this point.

The process to parameterize cognitive feedback is model and application specific. Unlike standard constraint-based user interfaces that are described in Myers et al. [18], new visualizations in the bi-directional pipeline do not simply result from fixing adjustments in 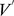 and configuring what remains in the visualization accordingly. Rather, we learn from 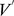 how we might adjust display-generating parameters that would impact the entire display jointly. Careful thought is needed to interpret and quantify cognitive feedback in a form that *both* captures the users' intent (reasons for injecting the cognitive feedback) and is compatible with the model. In the next section, we highlight what needs to be considered when parameterizing feedback and, in subsequent sections, we provide examples within the context of case studies.

#### 2.2.3 Parameterizing Feedback

In both the original and bi-directional visualizations pipelines, the visualizations depend upon a model 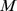 with inputs, data 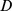 and parameters 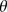. If we consider the data 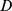 to be given (e.g., we do not transform nor filter the data), visualizations can only change when we alter specifications for 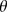. Thus, in some sense, all visualizations rely on potentially tunable parameters 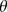. Within the context of V2PI, we parameterize feedback that is communicated by 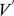 to tune specifications for 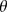; i.e., we use 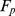 to adjust the model parameters from an original setting, 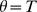, to a setting that accounts for feedback, 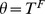. In turn, new visualizations rely on the model 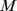, data 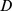, and expert-adjusted parameter specifications 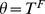. For example, some models 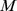 rely on an optimization procedure to set 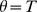 and visualize data 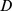. Based on 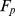, we might adjust the procedure which will subsequently result in calculating 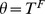.

The challenge is formulating 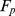 from 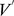 so that we can specify 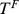. Our solution is two fold. First, we solve an inverse problem in that we estimate a value for 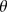 that would result in either the adjusted display 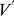 or the adjusted observations within 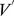. This solution is 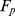, a parametric interpretation of 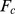. Second, we take a weighted average of 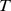 and 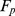 to set 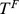,
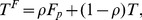
(1)


where 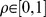 and 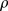 reflects the weight users want to place on their judgements relative to the current visualization, e.g., when 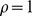, 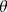 is specified entirely by expert judgement in that 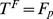. The choice to take a weighted average of 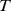 and 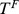 is both flexible and justifiable theoretically when 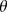 is assessed using Bayesian methods [19]. If users are unclear about weight 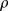, they may apply parametric interaction to observe how their feedback impacts a visualization by slowly transitioning 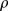 between 0 and 1.

We have mentioned several times that V2PI may occur in sequence; i.e., the bi-directional pipeline may repeat several iterations before a user feels satisfied with the data exploration. With each injection of cognitive feedback, a parametric form is derived and a new visualization is created. To convey this mathematically, consider the 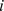
th execution of V2PI such that
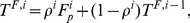
(2)


where 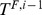 represents the specification for 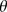 that created the visualization which was adjusted for the 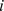
th iteration and 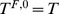 (the original specification for 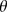). There is no notion of convergence when considering V2PI. Users choose to stop iterating when the data visualizations make sense. In some cases this means that users may stop when a particular structure in the data appears or, in other cases, when users assess the data from multiple perspectives (based on multiple implementations of V2PI) and simply feel comfortable with the data exploration. For the sake of being clear about V2PI, we exemplify it and the bi-directional pipeline in the next section using case studies that fall into the former category.

## Results

We provide two case studies that rely on either PCA or MDS to demonstrate the development and use of V2PI. PCA and MDS are similar (in fact, under some conditions, the same) in that each produces a spatialization of data for which the relative pairwise distances between observations has meaning; observations that appear close or far apart in visualizations are similar and different, respectively, in the high-dimensional data spaces. Thus, each case study allows users to explore data and adjust the coordinates of two or more observations (hence change the relative distances between points) to communicate cognitive feedback in the bi-directional visualization pipeline. However, PCA and MDS can differ by the way they learn the low-dimensional relative distances between observations. In turn, the methods we use to interpret and parameterize the cognitive feedback in the visualizations are different.

We begin each case study with a description of data and theoretical details of the analytical procedures, PCA or MDS. Then, we use the steps of the bi-directional pipeline to guide our discussions. We develop V2PI based on the parameterization of one form of cognitive feedback per example.

### 3.3 Case Study 1: PCA

#### 3.3.1 Data

The bi-directional pipeline and V2PI fosters data exploration and has the potential to reveal structure (when it exists) in data spatializations, such as clusters. To begin an exploration, experts often use what they know about the data. However, what they “know,” may be incomplete or reflect mere conjectures. For example, in genetic analyses, biologists might know the pathways to which some genes belong, but not all; or, to assess voting tendencies, political analysts might know the party affiliations of some voters, but not all. For such cases, it is reasonable to take semi-supervised analytical approaches to assess data and infer the global data structures. In this section, we use simulated data to emulate such scenarios.

**Figure 4:**
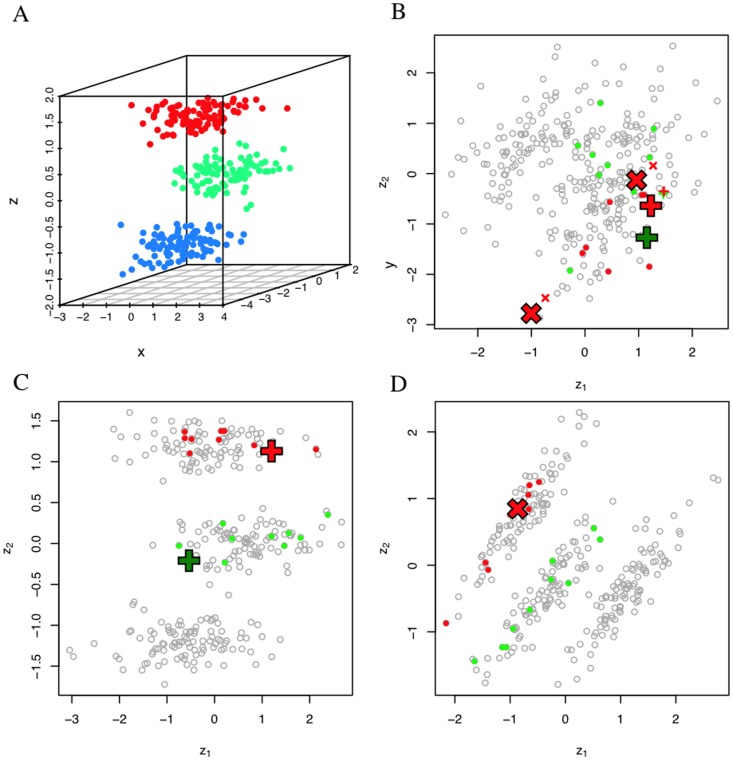
V2PI with PCA. Figure A displays the simulated data in three dimensions. Observations in red, green, and blue denote groups 1, 2, and 3 respectively. Figure B displays the PCA projection of the simulated data with 20 observations (that were selected at random) highlighted. Again, red and green points represent observations in groups 1 and 2 respectively. Figures C and D show updated displays after an adjustment to Figure B. Figure C is the result of moving points marked by ‘
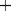
’ in Figure B apart and Figure D is the result of moving the points marked by ‘
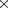
’ in Figure B together. Notice that both adjusted visualizations capture the clustering structure.

We simulated a 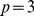 dimensional data set 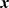 that contains 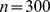 observations and three clusters, as shown in Figure 4A. Since we simulated the data, we have access to detailed information concerning the cluster assignments of each observation. However, we only reveal the cluster assignments for 20 of the 300 observations; ten observations were selected at random from clusters 1 and 2 each. To visualize these data we apply PCA and highlight the selected observations in Figure 4B. Notice in Figure 4B that observations from clusters 1 and 2 do not group. Rather, they are mixed in a seemingly random scatter within the remaining data. Based solely on the display, we cannot make judgements about, say, the number of clusters, size of clusters, and assignments of observations to clusters.

If we were willing to use the true classifications for the remaining 290 observations, we could define the clusters as a function of the dimensions in 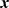 by using fully supervised learning strategies, such as a labeled version of PCA or linear discriminant analysis [20], [21]. However, we consider only what is known about the 20 highlighted observations in Figure 4B and we take a visual data exploration approach. In the sections that follow, we develop both the mathematical and computational machinery to apply V2PI and create new PCA visualizations. We start first by explaining the technicalities of PCA.

#### 3.3.2 Description of PCA

PCA is a deterministic, analytical procedure that relies on an optimal linear projector to reduce the dimension of a data set. Consider a center-shifted, 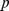-dimensional data set 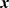 that contains observations 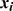 where 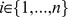; i.e., 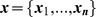 and 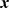 is 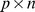. In our simulated example above, 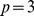 and 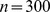. PCA relies on the solution for a 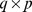 transformation matrix 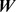, where 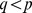, that maximizes the variance of a low-dimensional version of 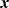 which we denote by 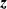.

To solve for 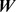, one option is to take the eigen-decomposition of the sample variance (of 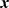), 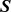, such that 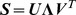, where 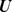 is 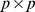 and contains the eigenvectors of 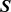, 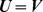, and 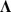 is a diagonal matrix that includes the ordered eigenvalues of 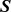 (e.g., the element in the first column and row of 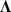 contains the largest eigenvalue of 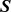). Since the eigenvectors that correspond to the two largest eigenvalues determine the two orthogonal directions that explain the most amount of variance in 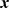, 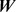 is assigned to equal the first two columns of 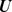,
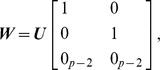



where 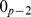 represents a 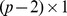 vector of zeros. Given 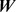, the calculation for 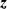 is straightforward,
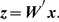
(3)


When 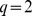, a PCA visualization simply plots 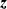 (e.g., Figure 4B) in a two-dimensional scatterplot. The axes of the plot are hard to interpret, but, fortunately, it is only the configuration of the points in the plot that matters. PCA spatializes observations so that the relative distance between them reflects their relative similarity in the dimensions most preserved. As defined by the current form of PCA, these dimensions are those with the largest variances. Alas, because of PCA's strict variance criteria and explicit assignment of 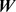, the spatialization can mask structures in data that do not correspond with variance. For example, the within-cluster variance is larger than the between-cluster variance in the data shown in Figure 4A. Thus, the clusters do not appear in 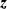 as plotted in Figure 4B, and despite knowing the presence and/or characteristics of the clusters, we cannot adjust 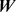.

In the next section, we transform PCA from a deterministic, dimension reduction algorithm to an expert guided projection method via V2PI and the bi-directional pipeline. We explain within the context of the data set described in Section 3.3.1. The goal is to allow experts to explore data from different perspectives using PCA methods so that the clusters may (or may not) be revealed. The advantage is that the interpretation of each data spatializations from the different perspectives is maintained (i.e., relative distances between observations reflect relative similarity), but structures that do not depend on variance have the potential to be discovered.

#### 3.3.3 PCA with V2PI

We start by applying PCA for Steps 1 and 2 in Figure 3. We derive 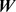 in accordance with Equation (3) and display 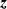 as we did in Figure 4B. For Step 3, experts participate in the data analysis by assessing and injecting feedback 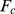 about the projection. Since the configuration of points has meaning in data spatializations, a natural surface-level interaction 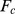 to parameterize is a reconfiguration of the points. Here, we develop V2PI so that users may re-configure the location of two observations; i.e., to create 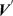, users may either drag two observations together or apart. The choice to drag observations together or apart depends upon expert judgment. If an expert believes two observations are similar in the high-dimensional space, but they appear distant in the visualization, the expert may drag the observations together. Whereas, if an expert believes two observations are different in the high-dimensional space, but they appear close together in the visualization, the expert may drag the observations apart. For example, in Figure 4B, a user may choose to drag two observations from cluster 1 (in red) together, two observations from cluster 2 (in green) together, or one observation from each cluster apart.

As a VA tool developer, we could have developed a more complex version of V2PI; e.g., allow users to move many observations. However, what we propose is still a viable form of V2PI and helps to convey the relative simplicity for how to use V2PI. Namely, experts need only have knowledge about the relationship between two observations to re-assess data from a different perspective. They do not need to have reliable judgments concerning, say, the dimensions in the data that define clusters; the number of clusters in the data set; nor the size of data clusters. Also, the methods can be extended to allow cognitive feedback with more than two observations. In fact, for the next case study, we do just that based on MDS (an analytical method that can re-produce PCA plots under some constraints); we allow users to move several observations to communicate cognitive feedback.

To parameterize 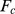 **Step 4** of the PCA bi-directional pipeline, we must a) determine a user's intent and b) represent it in a quantitative form that is compatible with PCA. When users drag observations together, the users are suggesting the need for a display that up-weights the dimensions for which the observations are similar and down-weights the dimensions for which they are different; whereas, when users drag observations apart, the users are suggesting the need for a display that up-weights the dimensions for which the observations are different and down-weights the dimensions for which they are same. For PCA, the dimensions that have relatively large and small weights are those with a high and low variances respectively-the transformation matrix 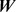 results from deterministic procedure based on the sample variance 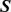. Thus, depending upon 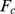, we re-weight the elements of variance matrix 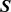 accordingly.

To do so, we derive a distance matrix as 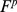 that is both indicative of the observation adjustments and similar in nature to a data variance matrix in that it is 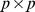 and semi-definite. We describe one procedure for deriving the distance matrix from 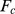 in File S1. Given 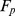, we take a weighted average as described in Section 2.2.3 to calculate 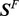,
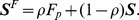



For a new visualization, we re-apply the PCA machinery; i.e., we determine the transformation matrix 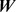 based on 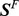 and re-calculate 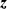 as defined by Equation (3).

We provide two adjusted PCA visualizations in Figure 4. Figures 4C and 4D are based on the cognitive feedback that two observations were dragged together and apart in step 3, respectively. Notice that regardless of the action taken for 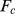, the adjusted figures display structure. In fact, from injecting information about the relationship between two observations, we learn from the updated view of the data that 1) the data include three clusters and 2) the cluster-assignments of every observation.

### 3.4 Case Study 2: MDS

#### 3.4.1 Data

In the previous case study, we used a simulated example to show how V2PI works. Now, we consider a more realistic dataset 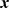 that describes 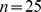 cities (Amherst, Ann Arbor, Atlanta, Atlantic City, Blacksburg, Bloomington, Boston, Chapel Hill, Charlotte, Chicago, Davis, Denver, Detroit, Fort Collins, Helena, Houston, Knoxville, Los Angeles, Miami, New York City, Reno, San Francisco, Seattle, Tucson, and Washington D.C.) based on ten variables: Latitude, Longitude, Income (median), Age (median), Population, Housing price (median), Population density, Highschool (percent over 25 who have completed high, school), Divorce rate (of those who have married), and Politics (percent voting for Obama versus McCain in 2008, county-wide). To add complexity to the data set, we append 20 noise variables; i.e., variables that were generated from Gaussian distributions with means zero and variances comparable to that of either the latitude or longitude variables.

**Figure 5:**
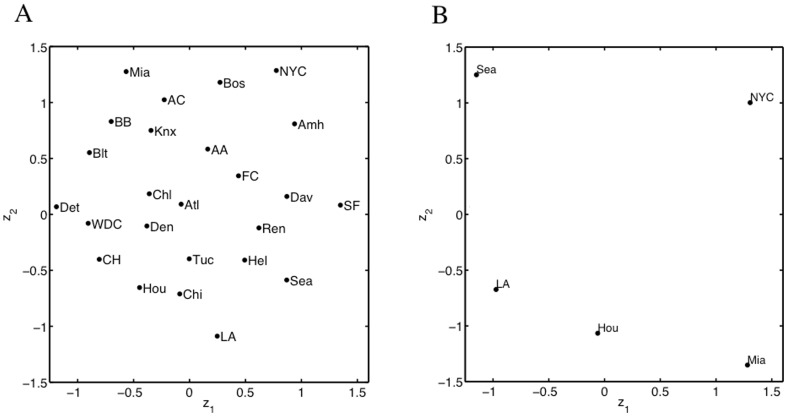
MDS view of the the city-data and an example of cognitive feedback. Figure A displays an Initial MDS view of the data set that describes 25 cities with 10 real variables and 20 noise variables. Figure B displays an example of cognitive feedback that arranges a set cities by relative geographic locations.

To visualize these data and assess varying structures in the data, we apply MDS as plotted in Figure 5A. To create this figure, all of the variables in the data set were weighted equally. Thus, the orientation we see of the data depends on both the real and noisy variable equally. A better orientation would isolate the important variables and down-weight those that are superfluous. For this reason, we develop V2PI for MDS.

#### 3.4.2 Description of MDS

In a classical MDS scheme [3], [22], the objective is to preserve pairwise distances between observations in low-dimensional representations of high-dimensional data. Using the same notation from the PCA example, we have a standardized data set 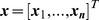 with 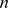 observations and 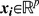 (for 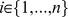). We aim to estimate a low-dimensional version of 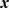 that we denote by 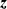, where 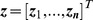, 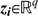 (for 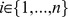), and 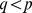. For the sake of visualization, 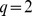 and, for our above example, 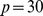. MDS solves for 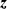 by minimizing the absolute difference between pairwise distances of observations in 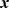 and 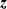,
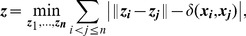
(4)


where 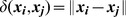, and 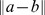 is a predefined norm of the distance between points 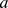 and 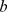. The right hand side of Equation (4) is typically referred to as a *stress* function, and the resolved minimum is called *the stress*. The norms used in Equation (4) will influence the MDS solution, if the distances themselves are sensitive to the norm under which they are computed. A common choice is the 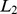 norm so that
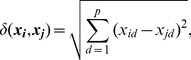
(5)


where 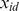 and 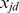 represent the 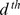 element in observations 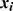 and 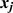 respectively. This choice is arbitrary and can be adjusted easily to accommodate other norms.

Similar to PCA, MDS produces a spatialization of the data 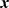 where relative distance between observations reflects their relative similarity. In fact, in the 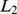 space, MDS will reproduce PCA visualizations. However, the explicit specification of a distance metric provides another means to parameterize feedback. For example, in the 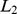 norm (Equation 5), all of the variables have equal importance or weight, even though we know that 20 variables are noise. Based on expert feedback, it makes sense to weight the variables in the distance metric so that only those that are relevant influence the visualization. Next, we develop V2PI for MDS by reparameterizing the distance metric.

#### 3.4.3 MDS with V2PI

To include expert judgments in MDS displays, we enable users to adjust (via cognitive feedback) a version of MDS known as Weighted Multi-Dimensional Scaling (WMDS) [23], [24]. Just as MDS, WMDS minimizes the stress function in Equation (4) to find a solution for 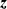. However, WMDS replaces 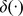 (the 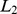 norm of two high-dimensional observations) with a weighted norm 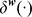,
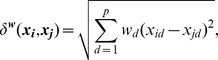



where 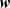 represents a pre-specified 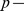
vector of dimension weights, 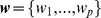, and 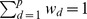. Given 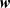, variables with large weights have more relevance than those with low weights in WMDS displays. Also, the MDS and WMDS solutions for 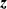 are identical when 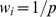 for each 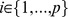.

Now, using the data from Section 3.4.1 and the bi-directional pipeline (Figure 3) as a guide for our discussion, we develop V2PI for MDS based on the WMDS machinery. We start by applying WMDS with 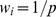 for **Steps 1** and **2** of the bi-directional pipeline. For **Step 3**, experts may reconfigure three or more observations to reflect a conjecture about the data and communicate cognitive feedback 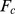. For example, since the data describe 25 cities, it might be helpful to visualize how the cities distribute geographically across the United States. However, we can see from Figure 5A that the cities are geographically misplaced; e.g., no matter how we rotate the display, Seattle, Miami, San Francisco and Houston will not orient geographically. Thus, as cognitive feedback 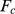, we enable MDS users to rearrange the locations of three or more cities to create 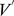. Figure 5B shows a possible orientation of five cities, Seattle (Sea), Los Angeles (LA), Houston (Hou), Miami (Mia), and New York City (NYC). Note that, similar, to PCA, there are a variety of surface-level interactions (which may eventually have a parametric interpretation) that users could perform with MDS visualizations. For this paper, we selected one.

The reason cities to do not map geographically is that the information in the variables, Latitude and Longitude, is masked by the remaining variables (both real and noise). Thus, for **Step 4** of the bi-directional pipeline, we parameterize 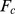 in the form of a weight vector that will up-weight the variables which seem to best explain 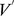 and down-weight those that do not. Let 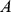 represent the set of 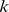 observations that were adjusted so that matrices 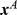 and 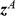 include only the high-dimensional and adjusted low-dimensional coordinates of the selected observations. To estimate new weights 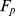, we solve the inverse problem; we solve for the weights that minimize the stress function based only data 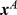 and 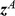. Explicitly, 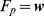, where
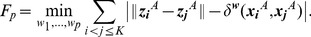
(6)


The solution 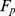 is found easily using a gradient search method [25] with the constraint 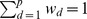. In our example, with cognitive feedback displayed in Figure 5B, the solution 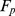 weights Latitude and Longitude by 0.47 and 0.52, respectively. The total weight of the remaining variables equals 0.01. Note, based on 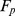, users may learn the variables that define the structure they find to be important. In this case, the users “learn” that the Latitude and Longitude explain their arrangement the observations because they have the largest weights. Although, we advocate the suppression of parametric information to users, weights have intuitive scales that are easy to interpret. That said, in an extremely high dimensional examples, creative, additional VA methods would be needed to provide this parametric information to users.

**Figure 6:**
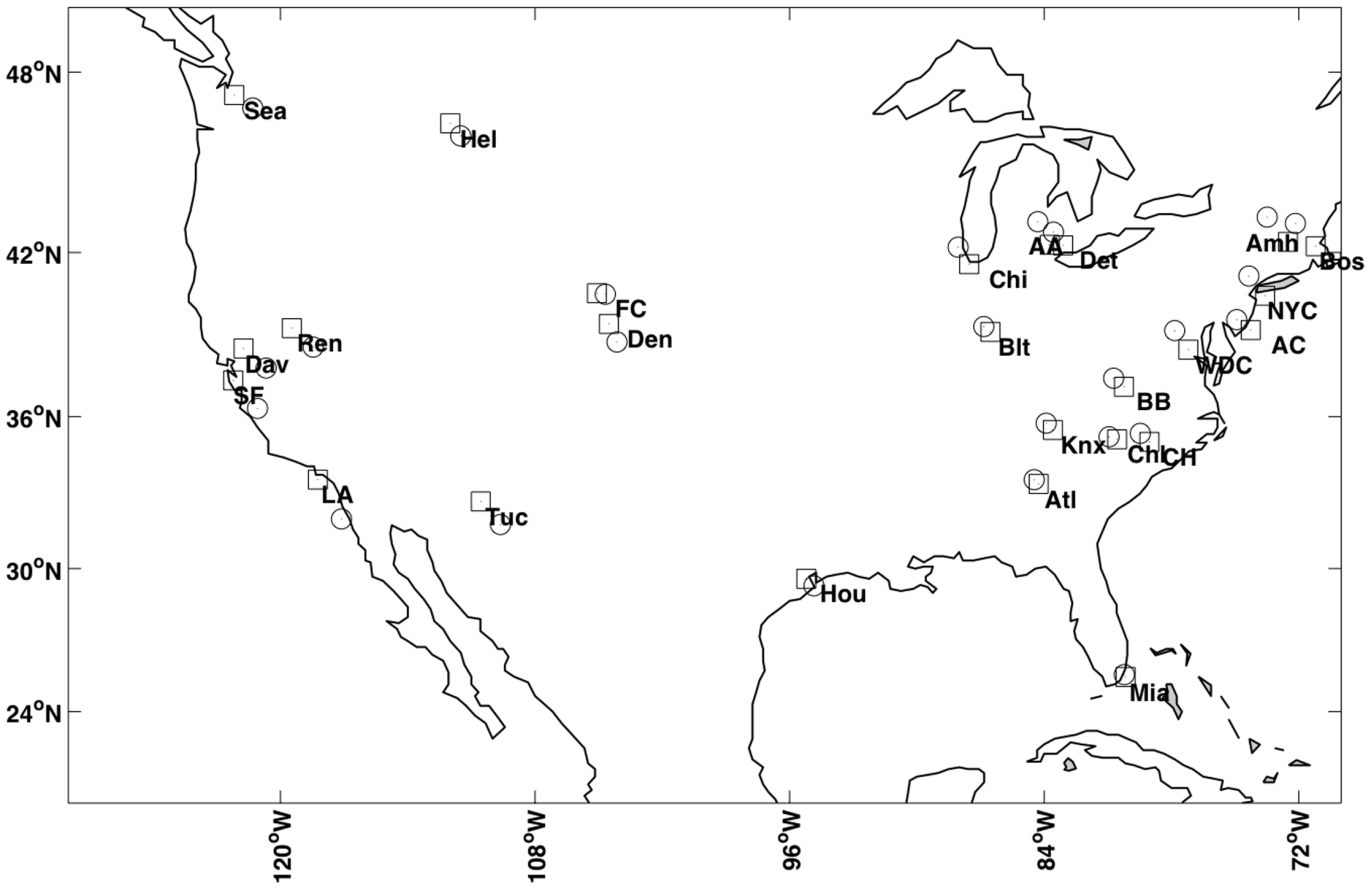
A visualization of the city-data that was updated by a parametric version of the cognitive feedback plotted in Figure 5B. The updated locations of the cities were stretched and rotated to overlay on a map of the United States. The symbols 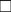 and 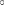 mark true and projected city coordinates by WMDS- V2PI. The estimated and true city coordinates are close.

To assess how the remaining data spatialize given cognitive and parametric feedback, we technically apply a weighted average as described in Section 2.2.3 so that
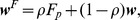



but we set 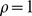 for this application of V2PI. In the standard MDS or WMDS procedures, the weight vector is pre-specified and set independently of the data, thus we do the same with V2PI. However, now, the weight vector is set according to user feedback. Subsequently, new data visualizations are created with the WMDS machinery and weight vector 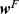. Figure 6 includes a new visualization of the data. Since Latitude and Longitude define the geographic locations of cities and we want to demonstrate the success of MDS with V2PI, we re-scaled and rotated the updated low-dimensional coordinates so that we could overlay them on a US map. On this map, we also include the true city coordinates. The user-guided visualization approximates the true map fairly well.

From our exploration of the data with V2PI and MDS, users a) visualize how the cities in the data set distribute across the United States from Figure 6 and b) learn from 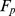 that Latitude and Longitude are the primary variables that explain the visual differences between the cities; e.g., Seattle and Miami are the furthest cities apart in Figure 6 because they differ the most in Latitude and Longitude. The data exploration could stop here, if users wanted. Or, users may reiterate the bi-directional visualization pipeline and inject more cognitive feedback to asses the data from another perspective. To show the latter is possible, we continue with the data exploration using V2PI in the next section.

#### 3.4.4 Continuation of MDS Data Exploration

Looking at Figures 5 and 6, we see that the data set includes major cities and college-towns. Suppose a user is unable to classify all of the observations (only a small set) and the user wants to learn which variables differentiate major cities from college towns. Figures 5A and 6 (two MDS visualizations of the data), do not help the user. Thus, the users apply V2PI again.

**Figure 7:**
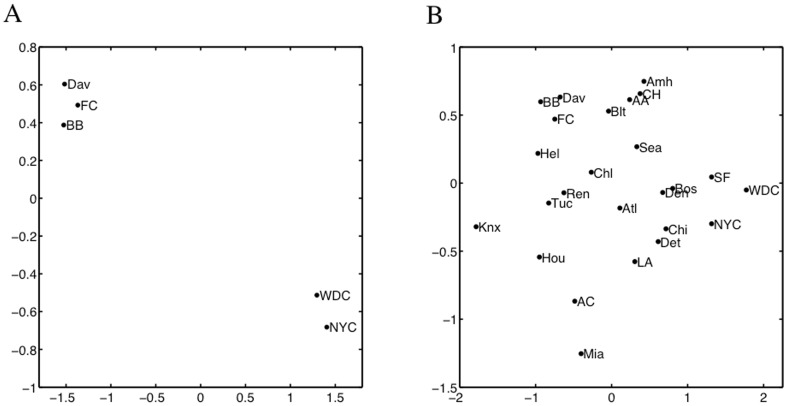
New cognitive feedback and updated view of city-data. Figure A plots another example of cognitive feedback that groups college towns separately from large cities. Figure B plots an updated visualization of the data that accounted for the feedback in Figure A.

For cognitive feedback 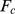, users move the cities about which the classification is known into two separate groups. Specifically, three college-towns, Blacksburg (BB), Davis (Dav), and Fort Collins (FC), are placed away from two cities, New York City (NYC) and Washington D.C. (WDC), to create 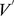 (Figure 7A). As previously shown, the methods are in place to learn weights 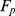 (the parameterized form 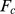), set 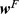 to 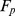, and create a new visualization (Figure 7B). According to 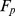, the selected observations differ most by Politics, Highschool, Age, and Population Density with weights 0.62, 0.22, 0.05, and 0.03, respectively. Also, based on proximity in Figure 7B, we see that Amherst (Amh), Ann Arbor (AA), Bloomington (Blt), and Chapel Hill (CH), are more similar to the selected college-towns (in the up-weighted variables) than the cities; and conversely, Boston (Bos), Chicago (Chi), Denver (Den), Detroit (Det), and San Francisco (SF), are more similar to the cities than the college-towns.

## Discussion

We applied V2PI using two common data visualization methods. In each example, constraints in the mathematical characterization of the data limited the utility of initial data displays; i.e., Figures 4A and 5A did not reveal expected nor meaningful structure. In turn, we included users in the visualization pipeline via V2PI to guide the mathematics and obtain visualizations worth assessing. The case studies provided successful examples and avoided some practical challenges that we discuss here.

V2PI does not guarantee the display of obvious data structure; V2PI only guarantees to portray user intent-as interpreted parametrically within the constraints of the display-generating model. For example, in the case studies, V2PI guaranteed new spatializations that reflected the users' judgements about the observations' pairwise relationships, as defined by PCA and MDS. The improved depictions of the pairwise relationships were estimated by one, updated linear projection (by either PCA or MDS) of the data. Given different visualization methods, the pairwise relationships might have updated differently. For example, V2PI could be developed for other visualization methods, including, Isomap, Generative Topographical Models, and Mixture PCA [26]–[28]. Such approaches characterize data spatially using non-linear methods and/or multiple visualizations. Had V2PI been in place with these methods, the updated displays of data in Figures 4C, 4D, 6, and 7B might have configured the observations differently.

It is important that an appropriate method is applied to assess data visually. V2PI, in its current form, only enables parameter adjustments within the chosen methods, not adjustments to the methods themselves. VA tools that enable users to switch the underlying analytical methods of visualization could be useful, as the selection depends upon both characteristics of the data and the analytical goal of the data exploration. In the case studies, there was little to no difference between PCA or MDS to visualize the datasets. We developed them to demonstrate differences in how we can conceptualize and parameterize feedback. Had either dataset within the case studies included outliers or non-linear relationships between observations neither PCA nor MDS (based on the 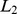 norm) would have been appropriate; non-linear methods, such as Isomap [26] or Generative Topographical Mapping [27] might be better. Also, there are several visualization methods that do not use geographic metaphors to display information. For example, cluster algorithms or network models may plot dendograms or directed graphs to group and link one or more observations together. Albeit, clusters were revealed in the first, PCA case study, but PCA is not formally a cluster-discovering algorithm; the cluster assignments were up to the user (which has advantages and disadvantages). If a user wants to formally classify observations, an appropriate analytical method should be applied and V2PI can be developed accordingly.

Crucially, the selected analytical method determines both the ways by which users can communicate cognitive feedback and how it is parameterized. This was discussed in Section 2.2. In the case studies, we presented only one form of cognitive feedback per analytical method; users adjusted the locations of either two or more observations. However, there are multiple forms of cognitive feedback that are applicable to data spatializations, including filtering, querying, and annotating, that can be parameterized. Future work of this research includes the implementation of user studies to learn the various forms in which it is natural for users to convey cognitive feedback based on a variety of visualizations.

In such user studies, we would also assess how analysts learn to use VA tools with V2PI and quantify what they gain from V2PI. Different analytical methods and datasets may result in visualizations that vary in difficulty to interpret. Only once users understand the meaning of the displays, can they effectively inject feedback and make sense of data. Thus, V2PI is most advantageous when users can learn how to interpret displays and interact with them more efficiently than understand the display-generating parameters. For example, in the first case study with PCA, it is reasonable to argue that users can assess and compare the relative differences between observations in visualizations with less effort than interpret the meaning of variance matrices, eigenvalues, and eigenvectors. Also, with V2PI, users can compare observations with PCA from varying perspectives to discover multiple structures or relationships in data easier than without V2PI. User studies that evaluate how well analysts understand PCA visualizations and compare what analysts learn using PCA with and without V2PI would likely support this argument. We envision a study that asks several questions about dataset(s) that analysts would be challenged to answer using what they know and VA tools with and without V2PI capabilities. The answers to the questions and the time it takes to answer the questions would illuminate the ease at which the analysts interpret the visualizations and the utility of the V2PI.

As with any user study, the dataset(s) that analysts are requested to assess is an important experimental-design element and may impact study results for several reasons. For example, datasets about which some users have prior knowledge and others do not will confound study findings. To evaluate V2PI, the size of datasets (in both the number of rows and columns) is also important to consider. Large datasets may impact the interpretability of some visualizations and computation time. In Figure 4, the dataset is small enough so that the points (i.e., observations) are distinguishable; distance between many observations was clear enough to inject feedback. Given millions of overlapping observations, this might not be the case. Also, with V2PI, real-time visualization updates enable users to explore data in parallel with them learning or thinking about the data. Yet, V2PI, as any analytical method, is limited by computational feasibility and efficiency. Thus, it is important to select datasets for studies that meet the constraints of the analytical method. Or, conversely, select/develop analytical and updating methods that can scale with data size. Both PCA and MDS (for certain distance metrics) are scalable.

## Conclusion

In this paper, we discussed two fundamental concepts: the bi-directional visualization pipeline and V2PI. When we combine the two, we have a visualization scheme that enables experts to explore data from multiple perspectives without understanding the display-generating models. Since users do not need to understand the mathematical underpinnings of visualizations, they are free to build upon their knowledge base and merge their expertise with the information in data instantly. That is, they may have an opportunity to learn and interact with a dataset directly in its visual domain-the domain in which experts host their expertise and intuition.

An important feature of the bi-directional pipeline is that users receive and distribute information, thus both expert judgement and standard datasets are valid components of quantitative analyses (that underlie data visualizations). The use of each component is not particularly novel when analyses are constructed within the Bayesian paradigm. Bayesian models combine prior distributions that may represent subjective, expert-driven information, with likelihoods to formulate inferences. However, the bi-directional pipeline 1) does not require formal probabilistic specifications to operate; 2) enables experts to communicate their judgements via data visualizations; and 3) allows experts to inject their judgements during multiple stages of data analyses. Experts have multiple opportunities to recall, include, and reflect upon their judgements in analyses by adjusting visualizations at each iteration of the bi-directional pipeline.

We exemplified the use of the bi-directional visualization pipeline and V2PI within two case studies. For each, a projection method was used to spatialize data in two dimensions and we described a unique approach to implement V2PI. The approaches differed due to the subtle differences in the projection methods. For visualizations that do not rely on linear projections, the bi-directional visualization pipeline and V2PI may still apply. However, V2PI has practical limitations that were discussed when we reflect on the case studies, including the selection of visualization methods that are appropriate for both the data and expert, the necessary learning curve for using V2PI, and computational feasibility. Each limitation is addressable with careful thought and flexible VA tools.

That said, successful interactions with data via visualizations rely upon the development of VA tools that support V2PI. The best VA tools are intuitive and accessible to users with varying levels of expertise. In this paper, we did not mention aesthetic aspects of VA tools that need to be considered for human cognitive purposes. Rather, we discussed the analytical mechanics needed in tools that enable V2PI.

## Supporting Information

File S1 **Parameterizing feedback for PCA.**
(PDF)
