# Supplementary material for: Visual to Parametric Interaction (V2PI)
Source: PLoS One. 2013 Mar 20;8(3):e50474. doi: 10.1371/journal.pone.0050474 (PMC3609854; doi:10.1371/journal.pone.0050474)
Supplement: File S1 — Parameterizing feedback for PCA. (PDF) [file pone.0050474.s001.pdf]

This documents provides supporting information for the paper *Visual to Parametric Interaction (V2PI)* by Leman, House, Maiti, Endert, and North. Refer to the paper for definitions and notation.

S1: Parameterizing Feedback for PCA

In Section 3.3.3 of *Visual to Parametric Interaction (V2PI)*, we develop V2PI for PCA. Users are allowed to move observations in a low-dimensional space as cognitve feedback, F_c. To parmeterize the feedback, we derive a distance matrix F^p that is similar in nature to a data variance matrix in that it is $p \times p$ and semi-definite. A PCA based on this distance matrix will reflect features of F_c.

Since we can either move observations together or apart, we define distances matrices for each move-type. Let Σ_a and Σ_t represent the distance matrices for the apart and together move-types respectively. We then take a weighted average of each to calculate the F_p,

$$F_p = \nu\Sigma_a + (1-\nu)\Sigma_t,$$

where $\nu \in [0,1]$. The derivations of ν, Σ_a, and Σ_t are deterministic functions of numerical summaries from the cognitive feedback.

Derivation of ν: This parameter reflects the degree to which experts move observations together or apart. Thus, to determine ν we calculate the ratio of the distances between observations j and k in the two dimensional display before and after the injection of cognitive feedback:

$$\tilde{F} = \frac{||\tilde{\boldsymbol{z}}_{\boldsymbol{j}} - \tilde{\boldsymbol{z}}_{\boldsymbol{k}}||_2}{||\boldsymbol{z}_{\boldsymbol{j}} - \boldsymbol{z}_{\boldsymbol{k}}||_2},$$

where $\boldsymbol{z}.$ and $\tilde{\boldsymbol{z}}.$ reference respectively the before and after coordinates of observations j and k. When observations are consolidated, $\tilde{F}$ is less than one and greater than one otherwise. Since $\nu \in [0,1]$, we use $\tan^{-1}(\cdot)$ to transform $\tilde{F}$ accordingly; $\nu = 2\tan^{-1}(\tilde{F})/\pi$.

Derivation of Σ_a: When observations are separated, we learn that the dimensions reflected poorly in the display need to be up-weighted. Thus, we start by quantifying the degree to which dimensions are unexplained in the visualization. Let $\boldsymbol{d}$ represent the $p \times 1$ raw discrepancy vector between observations j and k,

$$\boldsymbol{d} = |\boldsymbol{x}_{\boldsymbol{j}} - \boldsymbol{x}_{\boldsymbol{k}}| \tag{1}$$

and d_l refer to the observation discrepancy in dimension l. We define the projected discrepancy as

$$\boldsymbol{d}^{(p)} = |\boldsymbol{W}(e_l d_l)|$$

where e_l is the lth unit vector. The only nonzero element in $d^{(p)}$ is the lth element, $d_l^{(p)}$. If the raw and projected discrepancies are similar (or different) in dimension l, the visualization characterizes dimension l well (or poorly). To quantify this relationship, we choose to calculate the percent of *unexplained* discrepancy for each dimension l, $U_l = (1 - d_l^{(p)}/d_l)$, and define $d_l^{(u)}$ as

$$d_l^{(u)} = d_l U_l.$$

Collectively, we define the vector of unexplained discrepancies as $\boldsymbol{d}^{(u)} = [d_1^{(u)}, ..., d_p^{(u)}]$.

To define the projection plane that is reflective of the injected feedback, we need two perpendicular vectors which we denote as $\boldsymbol{\kappa}^{(u)}$ and $\boldsymbol{\kappa}^{(o)}$. We define $\boldsymbol{\kappa}^{(u)}$ based on $\boldsymbol{d}^{(u)}$ where $\boldsymbol{\kappa}^{(u)}$ is the normalized sum of $\boldsymbol{d}$ and $\boldsymbol{d}^{(u)}$,

$$\boldsymbol{\kappa}^{(u)} = \frac{\boldsymbol{d} + \boldsymbol{d}^{(u)}}{||\boldsymbol{d} + \boldsymbol{d}^{(u)}||_2}.$$

This vector, in comparison to the first principal component, has the potential to double the weight of under-represented dimensions in the visualisation. For example, if two dimensions have similar residual variances, this vector will add more weight to directions that were not previously explored in the contested visualization. To define the other vector, $\boldsymbol{\kappa}^{(o)}$, we select the direction that is both perpendicular to $\boldsymbol{\kappa}^{(u)}$ and explains the most amount of variance in $\boldsymbol{x}$.

The last step is to combine vectors $\boldsymbol{\kappa}^{(u)}$ and $\boldsymbol{\kappa}^{(o)}$ and determine Σ_a. To do so, we take the outer-produce of $\boldsymbol{\kappa}^{(u)}$ and $\boldsymbol{\kappa}^{(o)}$,

$$\Sigma_a = [\boldsymbol{\kappa}^{(u)}\ \boldsymbol{\kappa}^{(o)}][\boldsymbol{\kappa}^{(u)}\ \boldsymbol{\kappa}^{(o)}]'.$$

The spectral decomposition of Σ_a includes, by definition, eigenvectors $\boldsymbol{\kappa}^{(u)}$ and $\boldsymbol{\kappa}^{(o)}$ with corresponding eigenvalues of one.

Derivation of Σ_t: When observations are consolidated, we learn explicitly that, in an ideal display of the data, the adjusted observations should appear close together. One way to obtain an ideal display is to project the data in the direction defined $\boldsymbol{d}$ in Equation (1). This projection will map the adjusted observations to the exact same coordinates. To determine the vectors that define the projected plane, we solve for vectors $\boldsymbol{\kappa}^{(1)}$ and $\boldsymbol{\kappa}^{(2)}$ that are both perpendicular to one another and $\boldsymbol{d}$. Thus, the solution to the following system of equations determines a useful projection plane that is reflective of observations j and k together:

$$0 = \boldsymbol{d}'\boldsymbol{\kappa}^{(1)} = \boldsymbol{d}'\boldsymbol{\kappa}^{(2)} = \boldsymbol{\kappa}^{(1)'}\boldsymbol{\kappa}^{(2)}.$$
